# Preoperative Detection of Liver Involvement by Right-Sided Adrenocortical Carcinoma Using CT and MRI

**DOI:** 10.3390/cancers13071603

**Published:** 2021-03-31

**Authors:** Alice Kedra, Anthony Dohan, Sébastien Gaujoux, Mathilde Sibony, Anne Jouinot, Guillaume Assié, Lionel Groussin Rouiller, Rossella Libé, Jérôme Bertherat, Philippe Soyer, Maxime Barat

**Affiliations:** 1 Department of Diagnostic and Interventional Imaging, Hôpital Cochin, Assistance Publique—Hôpitaux de Paris, 75014 Paris, France; anthony.dohan@aphp.fr (A.D.); philippe.soyer@aphp.fr (P.S.); maxime.barat@aphp.fr (M.B.); 2 Faculté de Médecine, Université de Paris, 75006 Paris, France; sebastien.gaujoux@aphp.fr (S.G.); mathilde.sibony@aphp.fr (M.S.); anne.jouinot@aphp.fr (A.J.); guillaume.assie@aphp.fr (G.A.); lionel.groussin@aphp.fr (L.G.R.); jerome.bertherat@aphp.fr (J.B.); 3 Department of Surgery, Hôpital Cochin, Assistance Publique—Hôpitaux de Paris, 75014 Paris, France; 4 Department of Pathology, Hôpital Cochin, Assistance Publique—Hôpitaux de Paris, 75014 Paris, France; 5 Department of Oncology, Hôpital Cochin, Assistance Publique—Hôpitaux de Paris, 75014 Paris, France; 6 Department of Endocrinology, Hôpital Cochin, Assistance Publique—Hôpitaux de Paris, 75014 Paris, France; rossella.libe@aphp.fr

**Keywords:** adrenocortical carcinoma, liver, hepatectomy, neoplasm, staging

## Abstract

**Simple Summary:**

The major prognosis factor of adrenocortical carcinoma is the completeness of surgery. Focal adrenocortical carcinoma bulge on computed tomography and adrenocortical carcinoma contour disruption on magnetic resonance imaging are highly reproducible signs. These signs are strongly associated with direct liver involvement by right-sided adrenocortical carcinoma on preoperative imaging. These findings may help surgeons plan surgical approach before resection and decrease the complication rate.

**Abstract:**

The major prognosis factor of adrenocortical carcinoma (ACC) is the completeness of surgery. The aim of our study was to identify preoperative imaging features associated with direct liver involvement (DLI) by right-sided ACC. Two radiologists, blinded to the outcome, independently reviewed preoperative CT and MRI examinations for eight signs of DLI, in patients operated for right-sided ACC and retrospectively included from November 2007 to January 2020. DLI was confirmed using surgical and histopathological findings. Kappa values were calculated. Univariable and multivariable analyses were performed by using a logistic regression model. Receiver operating characteristic (ROC) curves were built for CT and MRI. Twenty-nine patients were included. Seven patients had DLI requiring *en bloc* resection. At multivariable analysis, focal ACC bulge was the single independent sign associated with DLI on CT (OR: 60.00; 95% CI: 4.60–782.40; *p* < 0.001), and ACC contour disruption was the single independent sign associated with DLI on MRI (OR: 126.00; 95% CI: 6.82–2328.21; *p* < 0.001). Both signs were highly reproducible, with respective kappa values of 0.85 and 0.91. The areas under ROC curves of MRI and CT models were not different (*p* = 0.838). Focal ACC bulge on CT and ACC contour disruption on MRI are independent and highly reproducible signs, strongly associated with DLI by right-sided ACC on preoperative imaging. MRI does not improve the preoperative assessment of DLI by comparison with CT.

## 1. Introduction

Adrenocortical carcinoma (ACC) is a rare entity with an estimated incidence of 0.5–2 cases per million per year, and accounts for 0.04–0.2% of all cancer deaths in the USA [1,2]. ACCs are associated with a poor prognosis with a 5-year survival rate of approximately 40% [3].

Some clinical, pathological, and molecular features have been identified as prognostic factors in patients with ACC [4]. The European Network for the Study of Adrenal Tumors (ENSAT) staging system, taking into account tumor size, infiltration of surrounding adipose tissue, invasion of adjacent organs, positive lymph nodes, and distant metastases, provides an accurate stratification strongly associated with cancer-specific mortality [5]. Five-year survival of affected patients ranges from 60–80% for ACC confined to the adrenal space (stages I and II) to 35–50% for locally advanced ACC (stage III) and is much lower for metastatic ACC (stage IV), the latter being found in 20–40% of patients at the time of diagnosis.

When feasible, *en bloc* surgical resection is the only curative treatment of ACC [6]. Open surgery is the standard surgical approach, even though laparoscopic adrenalectomy can be a reasonable option, depending on surgeon experience, for tumors <6 cm without any evidence of local invasion. *En bloc* resection includes peritumoral/periadrenal retroperitoneal fat resection and, if invaded, resection of adjacent organs [7]. Vascular or adjacent organ invasion are not contraindications for surgery, since the only curative option remains a free-margin (i.e., R0) resection. However, it conveys high peri-operative morbidity and mortality [8]. Notably, hepatic resections including atypical resection, segmentectomy or formal right hepatectomy are sometimes necessary during right-sided ACC surgery. This is associated with specific perioperative management and induces higher perioperative morbidity that needs to be assessed in the benefit-risk balance of the procedure [9].

Preoperative cross-sectional imaging, including computed tomography (CT) and magnetic resonance imaging (MRI), provides features suggestive for malignancy such as ACC size >4 cm, tumor heterogeneity, irregular shape, and/or growing tumor. Imaging also has a main role in tumor staging and preoperative planning of surgical approach [7]. To our knowledge, very few studies have been performed to identify preoperative imaging signs of direct liver involvement (DLI) in right-sided ACC [10,11].

The aim of our study was to identify imaging signs on preoperative CT and MRI that are associated with DLI in patients with right-sided ACC.

## 2. Materials and Methods

### 2.1. Patients

**Figure 1:**
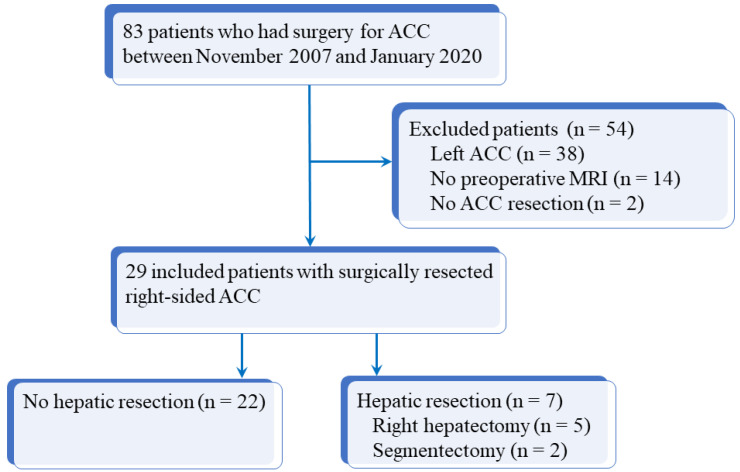
Flow chart of the study. ACC: adrenocortical carcinoma; MRI: magnetic resonance imaging.

Our institutional review board approved this study and informed consent was obtained from all patients. The database of our institution was queried from November 2007 to January 2020 inclusively to identify all consecutive patients who had surgery for ACC. Inclusion criteria were as follows: age > 18 years, history of operated and histologically proven right-sided ACC, and available preoperative imaging (both CT and MRI) within 3 months before surgery. Among 83 patients with operated ACC, 54 were excluded due to incomplete preoperative imaging (14 patients), left-sided ACC (38 patients), or unresected right-sided ACC (2 patients). Figure 1 shows the flow chart of the study. For the 29 patients included, the following data were recorded: age, sex, initial diagnosis date, European Network for the Study of Adrenal Tumors (ENSAT) stage (Supplemental Table S1), surgical and histopathologic findings (Weiss score, Ki67 rate), and overall survival defined as the duration of patient’s survival after surgery [12]. Initial characteristics of the 29 included patients are reported in Table 1. There were 8 men and 21 women with a median age of 46 years (q1 = 35; q3 = 59; range: 19–76 years). All patients had en bloc resection for right-sided ACC, according to international recommendations [13], including seven patients who had liver resection (five right hepatectomies and two segmentectomies).

All patients were followed-up at regular intervals with clinical, biological, and imaging examinations, with a minimal follow-up of 6 months.

### 2.2. Procedure and Imaging Protocol

All included patients had both preoperative abdominal CT and MRI performed less than 3 months before surgery. CT examinations were performed before and after intravenous administration of iodinated contrast material, with arterial (30 s) and portal venous (70 s) phases. MRI included at least the following sequences: diffusion-weighted imaging (DWI), T2-weighted half Fourier acquisition single-shot turbo spin-echo (HASTE), fat saturated (FS) T2-weighted BLADE, T1-weighted images (in- and out-of-phase), and dynamic multiphase contrast-enhanced sequences (Supplemental Table S2). Acquisition volume covered the right atrium to exclude right atrial thrombus [14].

### 2.3. Imaging Analysis

**Figure 2:**
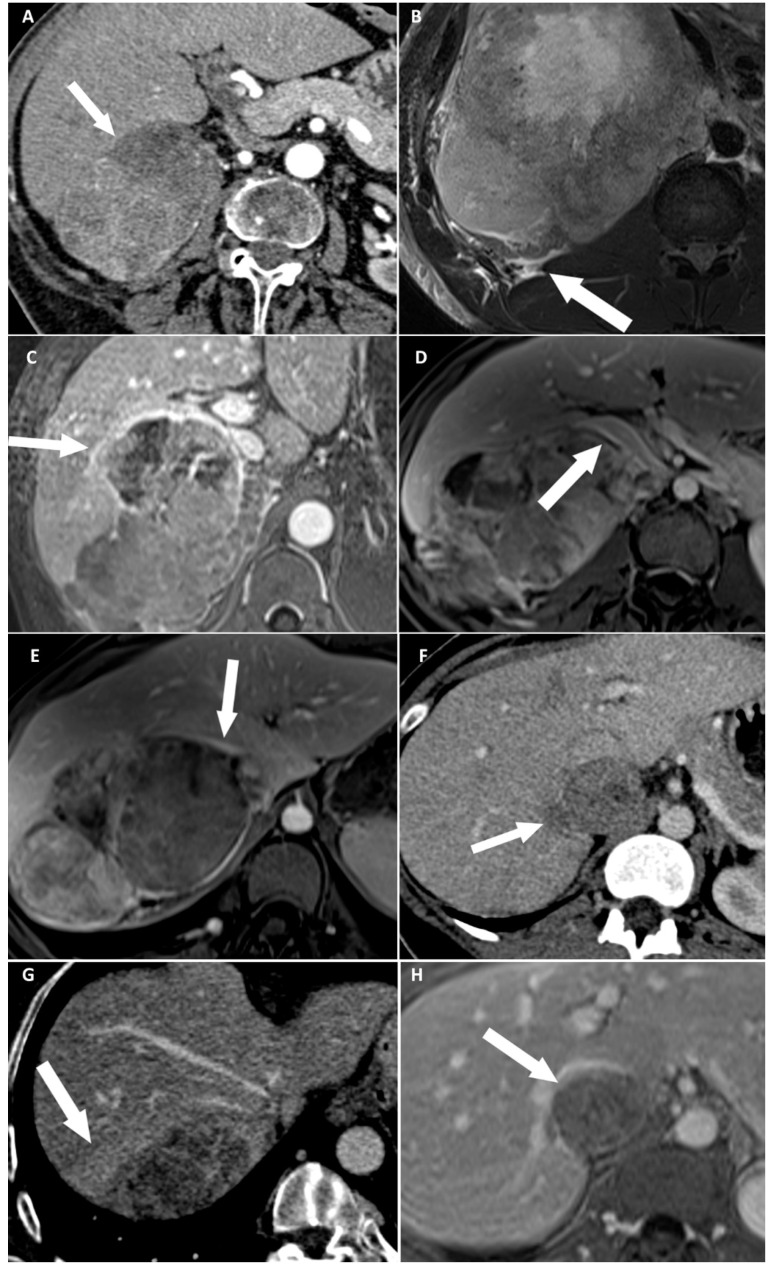
Categorical reading criteria. (**A**) A 76-year-old woman with a right-sided cortisol-secreting adrenocortical carcinoma (ACC) (Weiss score = 7, Ki67 = 13%), with direct liver involvement (DLI). CT image in the transverse plane obtained during the arterial phase following intravenous administration of iodinated contrast material shows disappearance of fat border between ACC and liver (arrow). (**B**) A 34-year-old man with a right-sided cortisol-secreting ACC (Weiss score = 9, Ki67 = 70%), with DLI. T2-weighted BLADE fat saturated (FS) image in the transverse plane shows periadrenal fat infiltration (white arrow). (**C**) A 57-year-old woman with a right-sided cortisol-secreting ACC (Weiss score = 9, Ki67 = 60%), associated with DLI. T1-weighted 3D volumetric interpolated breath-hold gradient-echo (VIBE) image in the transverse plane obtained during the venous phase following intravenous administration of gadoterate meglumine shows ACC contour disruption (arrow). (**D**) A 23-year-old woman with a right-sided cortisol-secreting ACC (Weiss score = 8, Ki67 = 50%), without DLI. T1-weighted VIBE image in the transverse plane obtained during the venous phase after intravenous administration of gadoterate meglumine shows macroscopic mass effect on inferior vena cava (arrow). (**E**) A 45-year-old woman with a right-sided ACC (Weiss score = 9, Ki67 = 16%), with DLI. T1-weighted 3D VIBE image in the transverse plane obtained during the venous phase after intravenous injection of gadoterate meglumine shows macroscopic mass effect on right hepatic vein (white arrow). (**F**) A 36-year-old woman with a right-sided noncortisol-secreting ACC (Weiss score = 7, Ki67 = 9%) associated with DLI. CT image in the transverse plane obtained during the venous phase following intravenous administration of iodinated contrast material shows focal ACC bulge (arrow). (**G**) A 55-year-old woman with a right-sided cortisol-secreting ACC (Weiss score = 6, Ki67 = 7 %), with DLI. CT image in the transverse plane obtained during the venous phase after injection of iodine based intravenous contrast agent shows periadrenal hepatic parenchyma enhancement (arrow). (**H**) A 36-year-old woman with a right-sided noncortisol-secreting ACC (Weiss score = 7, Ki67 = 9%) associated with DLI. T1-weighted VIBE image in the transverse plane obtained during the venous phase after intravenous administration of gadoterate meglumine shows ACC inclusion by hepatic parenchyma >180° (arrow).

**Figure 3:**
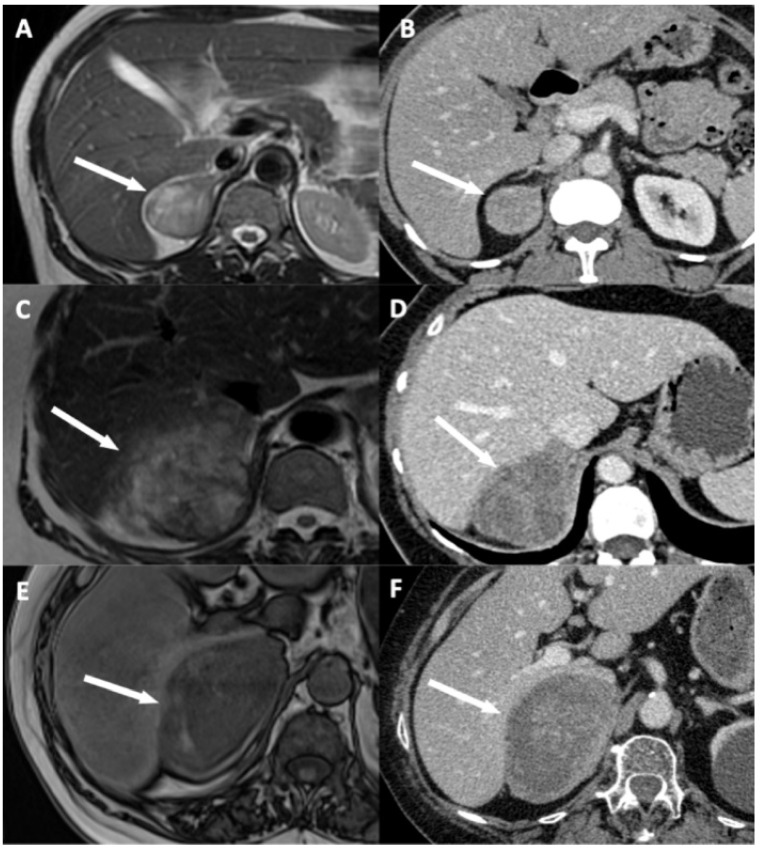
Illustration of disappearance of fat border between adrenocortical carcinoma (ACC) and liver. (**A**,**B**) A 53-year-old woman with a right-sided cortisol-secreting ACC (Weiss score = 3, Ki67 = 2%), without direct liver involvement (DLI). (**A**) T2-weighted half Fourier acquisition single-shot turbo spin-echo (HASTE) image in the transverse plane shows a visible and measurable fat border between ACC and liver (arrow). (**B**) CT image in the transverse plane obtained during the venous phase following intravenous administration of iodinated contrast material shows a visible and measurable fat border between ACC and liver (arrow). (**C**,**D**) A 57-year-old woman with a right-sided ACC (Weiss score = 7, Ki67 = 15%), with DLI. (**C**) T2-weighted HASTE image in the transverse plane shows a non-measurable (<1 mm) absent fat border between ACC and liver (arrow). (**D**) CT image in the transverse plane obtained during the venous phase following intravenous administration of iodinated contrast material shows a non-measurable (<1 mm) absent fat border between ACC and liver (white arrow). (**E**,**F**) A 73-year-old man with a right-sided ACC (Weiss score = 8, Ki67 = 20%), with DLI. (**E**) Unenhanced T1-weighted out-phase image in the transverse plane shows disappearance of the black boundary artifact normally visible at fat-water interfaces suggesting disappearance of fat border between ACC and liver (white arrow). (**F**) CT image in the transverse plane obtained during the arterial phase following intravenous administration of iodinated contrast material shows a non-measurable (<1 mm) absent fat border between ACC and liver (arrow).

**Figure 4:**
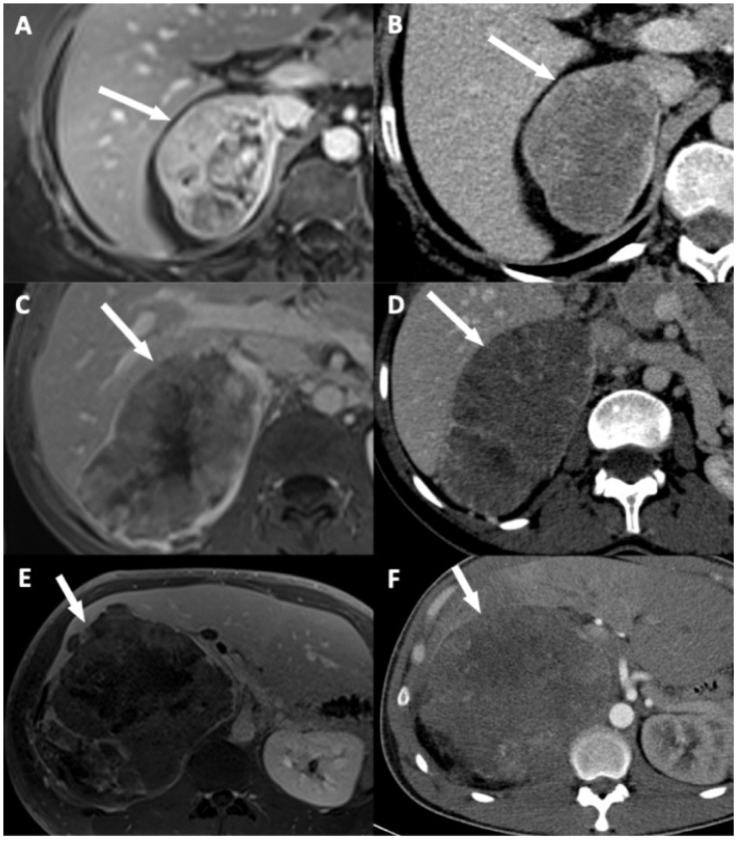
Illustration of adrenocortical carcinoma (ACC) contour disruption. (**A**,**B**) A 53-year-old woman with a right-sided ACC (Weiss score = 9, Ki67 = 40%), without direct liver involvement (DLI). (**A**) T1-weighted 3D VIBE image in the transverse plane obtained during the venous phase following intravenous administration of gadoterate meglumine shows a thin marginal enhancement of the lesion suggesting an intact adrenal capsule (arrow). (**B**) CT image in the transverse plane obtained during the venous phase following intravenous administration of iodinated contrast material t shows a thin marginal enhancement of the lesion suggesting an intact adrenal capsule (arrow). (**C**,**D**) A 34-year-old woman with a right-sided ACC (Weiss score = 9, Ki67 = 30%), with DLI. (**C**) T1-weighted 3D VIBE image in the transverse plane obtained during the venous phase following intravenous administration of gadoterate meglumine shows an adrenal capsular defect without any enhancement (arrow). (**D**) CT image in the transverse plane obtained during the venous phase following intravenous administration of iodinated contrast material shows an adrenal capsular defect without any enhancement (arrow). (**E**,**F**) A 34-year-old man with a right-sided ACC (Weiss score = 9, Ki67 = 70%), with DLI. (**E**) T1-weighted 3D VIBE image in the transverse plane obtained during the late phase (5 min) following intravenous administration of gadoterate meglumine shows an adrenal capsular defect without any enhancement (arrow). (**F**) CT image in the transverse plane obtained during the venous phase following intravenous administration of iodinated contrast material shows an adrenal capsular defect without any enhancement (arrow).

Two radiologists blinded to clinical outcomes (A.K. and M.B. with, respectively, 3 and 7 years of experience in abdominal imaging) independently reviewed all imaging examinations after anonymization on a picture-archiving and communication system (PACS) viewing station (DirectView^®^, 11.4.0.1253 sp1 version, Carestream Health, Rochester, NY, USA). For each examination, adrenal mass characteristics including dimensions on CT, and eight candidates imaging criteria of DLI were noted: (i) disappearance of fat border between ACC and liver; (ii) periadrenal fat infiltration; (iii) ACC contour disruption; (iv) macroscopic mass effect on inferior vena cava; (v) macroscopic mass effect on right hepatic vein; (vi) focal ACC bulge; (vii) periadrenal hepatic parenchyma enhancement; and (viii) ACC inclusion by hepatic parenchyma >180° (Figure 2, Figure 3 and Figure 4). Some of these signs have already been studied for locoregional invasion of ACC. Contrariwise, other signs have not been specifically analyzed for the assessment of DLI by ACC, but they have already been studied in other malignancies. Since the aim of our work was to identify new imaging signs associated with DLI by right-sided ACC, we tried to transpose these signs and their definitions for the study of locoregional extension of ACC. The fat border between ACC and liver was considered to be non-measurable and absent when <1 mm [10]. Periadrenal fat densification was defined on CT by a difference in attenuation values >10 Hounsfield units (HU) between normal retroperitoneal fat and peritumoral fat [15]. On MRI, it was characterized by hyperintense areas in the periadrenal fat on T2-weighted BLADE sequences. ACC contour disruption was defined on both CT and MRI by an adrenal capsular defect, which did not show any enhancement, contrary to a thin marginal enhancement of the lesion suggesting an intact adrenal capsule [11]. Macroscopic mass effect on inferior vena cava or right hepatic vein was defined by an anterior displacement of the vessel in association with direct contact with the tumor, with or without changes in caliber [16]. Focal ACC bulge was defined as focal protrusion of tumor into hepatic parenchyma [17]. Periadrenal hepatic parenchyma was considered enhanced by comparison with distant parenchyma if there was a measurable attenuation difference on portal phase images, with a threshold of 20 HU [18]. Inclusion by hepatic parenchyma was considered on axial images when the liver parenchyma surrounded the tumor over its half-circumference (Table 2).

### 2.4. Standard of Reference

At surgery, DLI by ACC was considered in the presence of macroscopic tumoral capsular rupture associated with invasion of the Glisson capsule, macroscopic invasion of the fat border between the tumor and the liver, or disappearance of the anatomical space between the tumor and the liver, making a free-margin resection impossible to obtain [7].

After resection, all ACCs were graded using Weiss histopathologic criteria of malignancy, based on the evaluation of nine features: nuclear grade, mitotic rate, atypical mitotic figures, cytoplasm, diffuse architecture, necrosis, venous invasion, sinusoid invasion, and invasion of tumor capsule [19,20]. Histopathologic diagnosis of ACC was made if there were at least three of the nine malignancy criteria.

The definite diagnosis of DLI was made using surgical and histopathological findings. Patients who had no liver resection were considered as having no DLI by ACC.

### 2.5. Statistical Analysis

Statistical analysis was performed by using SPSS 23.0 software (SPSS, Chicago, IL, USA) and MedCalc, version 11.3.0 (MedCalc Software Ltd., Ostend, Belgium). Continuous variables were expressed as medians, interquartile ranges, and ranges. Qualitative variables were expressed as raw numbers, proportions, and percentages along with their 95% confidence intervals (CIs). Normality of distributions was assessed by using histograms and Shapiro–Wilk test. Continuous and categorical variables were compared by using Mann–Whitney and Fisher exact tests, respectively. Overall survival (OS) was estimated by using the Kaplan–Meier method. The log-rank test was used to compare survival curves. Overall survival was calculated from the date of surgery until death. Interobserver agreement for categorical variables was assessed using the weighted kappa (K) test, and K values were reported with their 95% CI [21].

Univariable analyses were conducted by logistic regression model to identify candidate features associated with DLI and to estimate odds ratios (ORs) and their 95% CIs. To consider confounders of DLI, a multivariable analysis was performed by using a logistic regression model with backward stepwise selection of covariates and with entering and removing limits of *p* < 0.10 and *p* > 0.05. Correlations between all variables were examined. In case of a strong correlation between two variables, one or another variable was included in the multivariable model. A multivariable model was built for CT features and a second for MRI features. ROC curve analysis was performed for these two models, and the area under the ROC curves (AUROC) was compared using the De long test to evaluate the added value of MRI. Significance was set at *p* < 0.05.

## 3. Results

### 3.1. Correlation of Survival of the Overall Cohort with DLI

The median OS was significantly lower in patient with DLI (25 months; q1 = 4, q3 = 33; range: 7–44 months) compared to those without DLI (110 months; q1 = 43, q3 = 60; range: 86–134 months) (*p* = 0.002). ENSAT stage >2, Ki67rate >20%, and Weiss score >7 were all significantly associated with lower OS (*p* < 0.001, *p* = 0.001, and *p* < 0.001, respectively).

### 3.2. Quantitative Findings

The dimensions of all ACCs were measured on preoperative CT. For patients without DLI, median tumor length was 80 mm (q1 = 49; q3 = 103; range: 21–170 mm), the median tumor width was 56 mm (q1 = 37; q3 = 67; range: 12–151 mm), and median tumor height was 62 mm (q1 = 51; q3 = 109; range: 19–235 mm). For patients with DLI, median tumor length was 105 mm (q1 = 82; q3 = 132; range: 63–160 mm), median width was 66 mm (q1 = 58; q3 = 99; range: 39–119 mm), and the median height was 101 mm (q1 = 81; q3 = 131; range: 45–179 mm). No differences in dimensions were found between patients with DLI and those without DLI. (Table 1).

### 3.3. Qualitative Findings

Interobserver agreement was perfect or nearly perfect for the three following signs: disappearance of fat border between ACC and liver, ACC contour disruption, and focal ACC bulge, with respective kappa values of 0.94 (95% CI: 0.87–1.00; *p* < 0.001) on CT and 0.85 (95% CI: 0.75–0.95; *p* = 0.001) on MRI, 1.00 (95% CI: 0.95–1.00; *p* < 0.001) on CT and 0.91 (95% CI: 0.82–1.00; *p* < 0.001) on MRI, 0.85 (95% CI: 0.75–0.95; *p* < 0.001) on CT and 1.00 (95% CI: 0.92–1.00; *p* < 0.001) on MRI (Table 3).

On CT and MRI, disappearance of fat border between ACC and liver was present in 3/22 patients (14%) without DLI and in 7/7 patients (100%) with DLI (*p* < 0.001). ACC contour disruption was present on CT and MRI for 1/22 patients (5%) without DLI, whereas it was present on CT for 7/7 patients (100%) with DLI, and on MRI, it was present in 6/7 patients (86%) with DLI (*p* < 0.001). For patients without DLI, focal ACC bulge was present in 2/22 patients (9%) on CT, and 3/22 patients (14%) patients on MRI. For patients with DLI, focal ACC bulge was present in 6/7 patients (86%) on CT and in 7/7 patients (100%) on MRI (*p* < 0.001) (Table 4 and Table 5).

### 3.4. Univariable and Multivariable Analysis

At univariate logistic regression analysis, three qualitative features and ENSAT stage were associated with DLI: disappearance of fat border between ACC and liver (Figure 2A and Figure 3), ACC contour disruption (Figure 2C and Figure 4), and focal ACC bulge (Figure 2F) (Table 1 and Table 6).

At multivariable analysis, focal ACC bulge was the single imaging sign independently associated with DLI on CT (OR = 60.00; 95% CI: 4.60–782.40). This sign yielded 86% sensitivity (6/7; 95% CI: 49–97%), 91% specificity (20/22; 95% CI: 72–98%), and 90% accuracy (26/29; 95% CI: 53–99%) for the diagnosis of DLI. On MRI, ACC contour disruption was the single independent sign associated with DLI (OR = 126.00; 95% CI: 6.82–2328.21). This sign yielded 86% sensitivity (6/7; 95% CI: 49–97%), 96% specificity (21/22; 95% CI: 78–99%), and 93% accuracy (27/29; 95% CI: 56–99%) for the diagnosis of DLI (Table 7).

The diagnostic capabilities of both CT and MRI were assessed using subgroup analysis restricted to patients presenting with at least focal ACC bulge and ACC contour disruption on CT or MRI. The same diagnostic capabilities were found for both focal ACC bulge and ACC contour disruption. Each sign, visible indifferently on CT or on MRI, had 100% sensitivity (7/7; 95% CI: 65–100%), 91% specificity (20/22; 95% CI: 72–97%), and 93% accuracy (27/29; 95% CI: 56–99%) for the diagnosis of DLI.

The diagnostic performances of different combinations of sign on CT and MRI was evaluated. ACC bulge or ACC contour disruption on CT yielded 100% sensitivity (7/7; 95% CI: 65–100%), 91% specificity (20/22, 95% CI: 72–97%), and 93% accuracy (27/29, 95% CI: 56–99%) for the diagnosis of DLI. Focal ACC bulge or ACC contour disruption on MRI yielded 100% sensitivity (7/7, 95% CI: 65–100%), 86% specificity (19/22, 95% CI: 67–95%), and 90% accuracy (26/29, 95% CI: 53–99%) for the diagnosis of DLI. The association of focal ACC bulge and ACC contour disruption on CT yielded 86% sensitivity (6/7, 95% CI: 49–97%), 95% specificity (21/22, 95% CI: 78–99%) and 93% accuracy (27/29, 95% CI: 56–99%) for the diagnosis of DLI. On MRI, the presence of both signs yielded 86% sensitivity (6/7, 95% CI: 49–97%), 95% specificity (21/22, 95% CI: 78–99%), and 93% accuracy (27/29, 95% CI: 56–99%) for the diagnosis of DLI (Table 7).

### 3.5. Added Value of MRI

**Figure 5:**
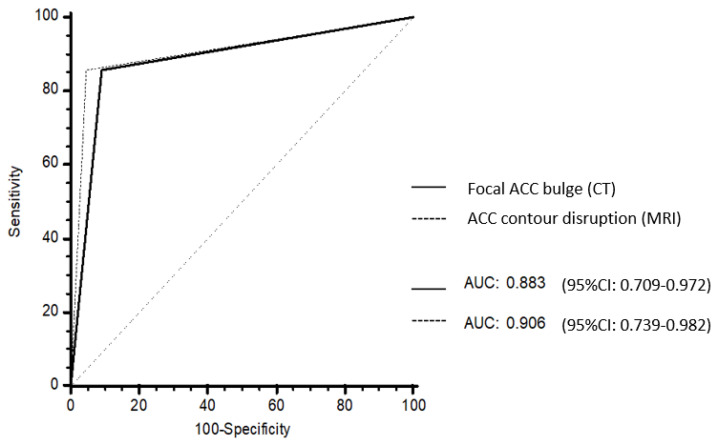
Receiver operating characteristic (ROC) curves of the CT and MRI models. Focal ACC bulge on CT had an area under the ROC curves (AUROC) of 0.883 (95% CI: 0.709–0.972). ACC contour disruption on MRI had an AUROC of 0.906 (95% CI: 0.739–0.982). There was no difference between AUROC at CT and AUROC at MRI (*p* = 0.838).

No differences in AUROC were found between focal ACC bulge on CT (0.883; 95% CI: 0.709–0.972) and ACC contour disruption on MRI (0.906; 95% CI: 0.739–0.982) (*p* = 0.838) (Figure 5).

## 4. Discussion

Preoperative prediction of DLI by right-sided ACC is mandatory in order to assess the benefit to risk balance of the procedure and tailor perioperative care. In the present study, we found that ACC contour disruption and focal ACC bulge on preoperative imaging were associated with DLI with high ORs and accuracies. Interestingly, we found that relatively small ACC may cause DLI, as the smallest lesion with DLI in our cohort measured 63 × 39 × 45 mm^3^, and there were no differences in terms of size between both groups.

Increased peritumoral hepatic parenchyma enhancement was identified as a sign of DLI in other studies. According to Tseng at al., hyperintensity of the liver parenchyma adjacent to gallbladder carcinoma is an indirect sign of DLI [18]. Perfusion hepatic disorders adjacent to tumor can be found in a variety of tumors, particularly when they affect a focal part of the hepatic parenchyma [22]. Some hypotheses have been suggested to explain this phenomenon. First, correlation with histopathological examination reveals that hepatic perfusion abnormalities indicate direct locoregional involvement of hepatic parenchyma by tumor or inflammatory reaction secondary to peritumoral sinusoidal dilatation and edema [23]. Finally, compressed or blocked portal blood vessels within the liver due to an extrinsic mass-effect can lead to a blood compensation via the hepatic artery, possibly producing a focal higher parenchyma enhancement [24]. However, in our study we did not find any significant association between hyperenhancement of peritumoral hepatic parenchyma and DLI.

In our study, patients who had liver resections had decreased OS compared to those who did not. This suggests that our study population is representative and similar to those of previous series. Indeed, ENSAT stage III ACCs whatever the organ invaded are known to have worst prognosis than lower stage ACCs and more specifically have poorer prognosis compared to those without hepatic involvement [5]. For selected patients, an aggressive surgical approach for ACC liver metastasis (ENSAT stage IV) is associated with long-term OS (5-year survival rate of 39% for operated metastatic patients) compared to conservative management (5-year survival of 15–20% for non-operated metastatic patients) [25].

In our study, we found that MRI does not improve the preoperative prediction of DLI by comparison with CT. The respective capabilities of these two imaging techniques for the diagnosis of adrenal tumors have already been studied. CT is usually considered as the most useful modality for identification and characterization of adrenal tumors. When lesions cannot be characterized adequately with CT or when CT is contraindicated, MRI remains an alternate option. However, MRI does not convey better spatial resolution, and this might explain why there is no difference for preoperative assessment of DLI between MRI and CT [16]. Nevertheless, there is no clear recommendation for the use of CT versus MRI for the diagnosis and preoperative staging of ACCs [13].

Our results demonstrate a high reproducibility of candidate signs, since the kappa values show nearly complete agreement between two radiologists with different levels of experience in abdominal radiology for the three most important signs. In addition, we used a strong standard of reference for the diagnosis of DLI because it was based on the association of surgical and histopathological criteria.

Our study has some limitations. First, its retrospective and monocentric design may limit generalizability. However, our center is a nationwide tertiary referral center for this rare disease, and the characteristics of our population are similar to those of other series reporting patients with ACCs [26,27]. Then, we included patients from 2007 to 2020, which may lead to interpretation bias toward imaging modalities. In fact, CT and MRI techniques evolved since 2007, especially concerning the quality of image and spatial resolution.

In conclusion, ACC contour disruption and focal ACC bulge are the two most accurate and reproducible signs associated with DLI on preoperative imaging. This may help surgeons plan a surgical approach before resections and decrease the complications rate. However, further prospective validation is needed to confirm these findings.

## Figures and Tables

**Table 1:** Initial characteristics of 29 patients with right-sided adrenocortical carcinoma.

	Patients without DLI n = 22	Patients with DLI n = 7	*p*-Value
**Age (years)**	40 (19–73) (q1 = 34; q3 = 58)	57 (34–76) (q1 = 43; q3 = 60)	0.33
**Sex (M/F)**	6/16 (38%)	2/5 (40%)	1.00
**Ki 67**	15/(2–95) (q1 = 5; q3 = 27)	45 (13–70) (q1 = 26; q3 = 63)	0.080
**Weiss score**	6 (3–9) (q1 = 4; q3 = 8)	9 (7–9) (q1 = 7; q3 = 9)	0.035
**Overall survival (months)**	110 (86–134) (q1 = 13; q3 = 82)	25 (7–44) (q1 = 4; q3 = 33)	0.002
**Tumor size**			
Length (mm)	80 (21–170) (q1 = 49; q3 = 103)	105 (63–166) (q1 = 82; q3 = 132)	0.15
Width (mm)	56 (12–151) (q1 = 37; q3 = 67)	66 (39–119) (q1 = 58; q3 = 99)	0.19
Height (mm)	62 (19–235) (q1 = 51; q3 = 109)	101 (45–179) (q1 = 81; q3 = 131)	0.15
**ENSAT stage** (median (range))	2 (1–4) (q1 = 2; q3 = 2)	3 (3–4) (q1 = 3; q3 = 3)	0.012
**I**	3	0	0.55
**II**	16	0	0.001
**III**	0	5	<0.001
**IV**	3	2	0.57

**Notes.** DLI: direct liver involvement; ENSAT: European Network for the Study of Adrenal Tumors; M: male; F: female. Qualitative variables are expressed as proportions; numbers in parentheses are percentages. Quantitative variables are expressed as medians; numbers in parentheses are ranges. Q1 means first quartile; q3 means third quartile.

**Table 2:** Categorical reading criteria used on different imaging modalities (CT and MRI) in 29 patients with operated right-sided ACC.

Imaging Sign	Imaging Modality	Analysis Criterion
Disappearance of fat border between ACC and liver	Portal phase CT T2 HASTE MRI (**)	Fat border between ACC and liver non-measurable (<1 mm)
Periadrenal fat densification	Portal phase CT T2 FSE MRI	ΔHU between periadrenal fat and normal retroperitoneal fat >10 HU Hyperintense areas in the periadrenal fat
ACC contour disruption	Portal phase CT Portal phase MRI	Measurable adrenal capsular defect without any enhancement
Macroscopic mass effect on inferior vena cava	Portal phase CT Portal phase MRI	Intrahepatic displacement of the vessel and direct contact with the tumor ± changes of its caliber
Macroscopic mass effect on right hepatic vein	Portal phase CT Portal phase MRI	Intrahepatic displacement of the vessel and direct contact with the tumor ± changes of its caliber
Focal ACC bulge	Portal phase CT Portal phase MRI	Focal and abrupt irregularity of ACC shape
Periadrenal hepatic parenchyma enhancement	Portal phase CT	ΔHU > 20 HU between periadrenal liver parenchyma and normal adjacent parenchyma
ACC inclusion by hepatic parenchyma > 180°	Portal phase CT Portal phase MRI	ACC surrounded by the liver parenchyma over its half-circumference

**Notes.** CT: computed tomography; MRI: magnetic resonance imaging; (**): main sequence for the evaluation; ACC: adrenocortical carcinoma; HASTE: half Fourier acquisition single-shot turbo spin-echo; FS: fast spin-echo; HU: Hounsfield unit.

**Table 3:** Interobserver agreement for categorical data in 29 patients with operated right ACC.

Categorical Data	CT			MRI		
	κ Value	95% CI	*p* Value	κ Value	95% CI	*p* Value
Disappearance of fat border between ACC and liver	0.94	0.87–1.00	<0.001	0.85	0.75–0.95	0.001
Periadrenal fat densification	0.37	0.20–0.53	0.021	0.37	0.20–0.53	0.02
ACC contour disruption	1.00	0.95–1.00	<0.001	0.91	0.82–1.00	<0.001
Macroscopic mass effect on inferior vena cava	0.88	0.80–0.96	<0.001	0.81	0.68–0.94	<0.001
Macroscopic mass effect on right hepatic vein	0.68	0.53–0.83	<0.001	0.72	0.58–0.87	<0.001
Focal ACC bulge	0.85	0.75–0.95	<0.001	1.00	0.92–1.00	<0.001
Periadrenal hepatic parenchyma enhancement	0.62	0.42–0.82	<0.001	0.37	0.18–0.57	0.04
ACC inclusion by hepatic parenchyma >180°	0.91	0.81–1.00	<0.001	0.81	0.68–0.94	<0.001

**Notes.** ACC: adrenocortical carcinoma; CT: computed tomography; MRI: magnetic resonance imaging; CI: confidence interval.

**Table 4:** Comparison of CT findings for independent categorical criteria between 22 patients without DLI and 7 patients with DLI.

	Patients without DLI n = 22			Patients with DLI n = 7			*p* Value
	Pr	%	95% CI	Pr	%	95% CI	
Disappearance of fat border between ACC and liver	3/22	14	5–33	7/7	100	65–100	<0.001
Periadrenal fat densification	1/22	5	1–22	5/7	71	36–92	<0.001
ACC contour disruption	1/22	5	1–22	7/7	100	65–100	<0.001
Macroscopic mass effect on inferior vena cava	12/22	55	35–73	6/7	88	49–97	0.20
Macroscopic mass effect on right hepatic vein	4/22	18	7–39	4/7	57	25–84	0.07
Focal ACC bulge	2/22	9	3–28	6/7	86	49–97	<0.001
Periadrenal hepatic parenchyma enhancement	2/22	9	3–28	1/7	14	3–51	>0.99
ACC inclusion by hepatic parenchyma >180°	5/22	23	10–43	1/7	14	3–51	>0.99

**Notes.** CT: computed tomography; DLI: direct liver involvement; Pr: proportion; CI: confidence interval; ACC: adrenocortical carcinoma.

**Table 5:** Comparison of MRI findings for independent categorical criteria between 22 patients without DLI and 7 patients with DLI.

	Patients without DLI n = 22			Patients with DLI n = 7			*p* Value
	Pr	%	95% CI	Pr	%	95% CI	
Disappearance of fat border between ACC and liver	3/22	14	5–33	7/7	100	65–100	<0.001
Periadrenal fat densification	0/22	0	0–15	5/7	71	36–92	<0.001
ACC contour disruption	1/22	5	1–22	6/7	86	49–97	<0.001
Macroscopic mass effect on inferior vena cava	13/22	59	39–77	6/7	86	49–97	0.37
Macroscopic mass effect on right hepatic vein	4/22	18	7–39	4/7	57	25–84	0.07
Focal ACC bulge	3/22	14	5–33	7/7	100	65–100	<0.001
Periadrenal hepatic parenchyma enhancement	3/22	14	5–33	1/7	14	3–51	>0.99
ACC inclusion by hepatic parenchyma >180°	6/22	27	13–48	1/7	14	3–51	0.65

**Notes.** MRI: magnetic resonance imaging; DLI: direct liver involvement; Pr: proportion; CI: confidence interval; ACC: adrenocortical carcinoma.

**Table 6:** Evaluation of the association between independent imaging findings and the actual status of DLI using logistic regression analysis in 29 patients with operated right ACC.

CT	OR (95% CI)	*p* Value
Disappearance of fat border between ACC and liver	+∞ (5.16–+∞)	<0.001
Periadrenal fat densification	39,8 (2.88–2532,19)	<0.001
ACC contour disruption	+∞ (9.96–+∞)	<0.001
Macroscopic mass effect on inferior vena cava	4.76 (0.45–252.19)	0.20
Macroscopic mass effect on right hepatic vein	5.55 (0.66–55.78)	0.07
Focal ACC bulge	45.26 (3.47–2813.61)	<0.001
Periadrenal hepatic parenchyma enhancement	1.63 (0.02–36.93)	>0.99
ACC inclusion by hepatic parenchyma >180°	0.58 (0.01–6.96)	>0.99
MRI		
Disappearance of fat border between ACC and liver	+∞ (5.16–+∞)	<0.001
Periadrenal fat densification	+∞ (5.02–+∞)	<0.001
ACC contour disruption	79.65 (5.01–5668.77)	<0.001
Macroscopic mass effect on inferior vena cava	3.98 (0.38–211.67)	0.37
Macroscopic mass effect on right hepatic vein	5.55 (0.66–55.78)	0.07
Focal ACC bulge	+∞ (5.16–+∞)	<0.001
Periadrenal hepatic parenchyma enhancement	1.05 (0.02–16.34)	>0.99
ACC inclusion by hepatic parenchyma >180°	0.46 (0.01–5.21)	0.65

**Notes**. DLI: direct liver involvement; ACC: adrenocortical carcinoma; CT: computed tomography; OR: odds ratio; CI: confidence interval; MRI: magnetic resonance imaging.

**Table 7:** Sensitivity, specificity, and accuracy of categorical CT and MRI variables for the prediction of direct liver involvement in 29 patients with operated right-sided adrenocortical carcinoma.

Imaging Finding	CT				MRI			
	Sensitivity 95% CI	Specificity 95% CI	Accuracy 95% CI	*p* Value	Sensitivity 95% CI	Specificity 95% CI	Accuracy 95% CI	*p* Value
Disappearance of fat border between ACC and liver	100 65–100	86 67–95	90 53–99	<0.001	100 65–100	86 67–95	90 53–99	<0.001
Periadrenal fat densification	71 36–92	96 78–99	90 53–99	<0.001	71 36–92	100 85–100	93 56–99	<0.001
ACC contour disruption	100 65–100	96 78–99	97 60–100	<0.001	86 49–97	96 78–99	93 56–99	<0.001
Macroscopic mass effect on inferior vena cava	86 49–97	46 27–65	55 24–83	0.20	86 49–97	41 23–61	52 21–81	0.37
Macroscopic mass effect on right hepatic vein	57 25–84	82 62–93	76 40–93	0.07	57 25–84	82 62–93	76 40–93	0.07
Focal ACC bulge	86 49–97	91 72–98	90 53–99	<0.001	100 65–100	86 67–95	90 53–99	<0.001
Periadrenal hepatic parenchyma enhancement	14 3–51	91 72–98	72 37–92	>0.99	14 3–51	86 67–95	69 34–90	>0.99
ACC inclusion by hepatic parenchyma > 180°	14 3–51	77 57–90	62 29–87	>0.99	14 3–51	73 52–87	59 26–85	0.65
Focal ACC bulge or ACC contour disruption	100 65–100	91 72–97	93 56–99	<0.001	100 65–100	86 67–95	90 53–99	<0.001
Focal ACC bulge and ACC contour disruption	86 49–97	95 78–99	93 56–99	<0.001	86 49–97	95 78–99	93 56–99	<0.001

**Notes.** CT: computed tomography; MRI: magnetic resonance imaging; CI: confidence interval; ACC: adrenocortical carcinoma.

## Data Availability

Data may be available upon request.

## Supplementary Materials

The following are available online at ‘https://www.mdpi.com/article/10.3390/cancers13071603/s1, Table S1: ENSAT Staging System. Table S2: MRI parameters.
