# Supplementary material for: Preoperative Detection of Liver Involvement by Right-Sided Adrenocortical Carcinoma Using CT and MRI
Source: Cancers (Basel). 2021 Mar 31;13(7):1603. doi: 10.3390/cancers13071603 (PMC8036813; doi:10.3390/cancers13071603)
Supplement: Supplementary file 1 [file cancers-13-01603-s001.pdf]

cancers

MDPI

Supplementary Material

Preoperative Detection of Liver Involvement by Right-Sided Adrenocortical Carcinoma Using CT and MRI

Alice Kedra [1,*], Anthony Dohan [1,2], Sébastien Gaujoux [2,3], Mathilde Sibony [2,4], Anne Jouinot [2,5], Guillaume Assié [2,6], Lionel Groussin Rouiller [2,6], Rossella Libé [6], Jérôme Bertherat [2,6], Philippe Soyer [1,2] and Maxime Barat [1,2]

Supplemental Table S1. ENSAT staging system.

ENSAT staging system	TNM classification
**I**	$T_1N_0M_0$
**II**	$T_2N_0M_0$
**III**	$T_{1–2}N_1M_0$<br>$T_{3-4}N_{0-1}M_0$
**IV**	$T_{1-4}N_{0-1}M_1$

Notes.ENSAT: European Network for the Study of Adrenal Tumors; TNM: Tumor Nodes Metastases. T1: tumor < 5cm; T2: tumor > 5 cm; T3: tumor infiltration into surrounding adipose tissue; T4: tumor invasion into adjacent organs; N0: absence of positive lymph nodes; N1: at least one positive lymph node; M0: absence of distant metastases and M1: presence of distant metastases.

Supplemental Table S2. MRI parameters.

	DWI	T2 HASTE	T2 BLADE FS	T1 in phase	T1 out of phase	T1 3D VIBE
Plane	Transverse	Transverse and coronal	Transverse	Transverse	Transverse	Transverse
Repetition time (ms)	6000	1000 - 1200	2540	120	120	4.05
Echo time (ms)	79	91 - 95	121	4.75	2.35	1.89
Flip angle (°)	90	150 - 160	147	70	70	14
Fat suppression	Yes	No	Yes	No	No	Spectral
Slice thickness (mm)	6 - 7	5	5	5 - 8	5 - 8	2.5
Intersection gap (mm)	1	0	0 - 1	0 - 1	0 - 1	0
Number of slices	26 - 32	32 - 38	30 - 32	40	40	88 - 112
Matrix size	130 - 296 × 156 - 296	200 - 260 × 320	256 × 256	260 × 320	260 × 320	320 - 384 × 234 - 240
Number of signal averages	3 - 4	1	1	1	1	1
Acquisition time (s)	180 - 200	130 - 145	92 - 144	140 - 190	140 - 190	190

Notes. MRI: Magnetic resonance imaging; DWI: Diffusion-weighted imaging; HASTE: Half-Fourier acquisition single-shot turbo spin-echo; BLADE: periodically rotated overlapping parallel lines with enhanced reconstruction; FS: Fat saturation; VIBE:Volumetric interpolated breath-hold gradient-echo. Repetition time depended on the duration of the breath cycle.
